# Generating the Best Available Science and Data to Inform Healthy Food Environment Policy

**DOI:** 10.1016/j.advnut.2024.100334

**Published:** 2024-11-06

**Authors:** Ashley J Vargas

**Affiliations:** National Institute of Environmental Health Sciences, Durham, NC, United States

In 2021 the United States President issued a Memorandum committing “…to make evidence-based decisions guided by the best available science and data… across every area of government” [1]. Policy change is a commonly recognized lever for addressing public health concerns, and policy research is situated near the furthest end in models of translational research [2,3]. This commitment of United States’ leadership to evidence-based policies is motivating to see as a career federal scientist, and it simultaneously reminds me that the biomedical research workforce writ large has been issued the unending challenge of generating “the best available science and data” if we are to work toward improving public health policy. This unending challenge seems particularly daunting in the field of nutrition and food science. Food itself is highly variable (e.g., by region vs. by storage/preparation method vs. by season harvested) and multidimensional (e.g., dietary patterns vs. food group vs. individual food vs. food components vs. nutrients), which then must be evaluated in the context of human biology (including nutritional status), which is highly variable and multidimensional, and then as research moves through the translational research framework [2,3], external influences (e.g., the social determinants of health, access to resources, the policy and regulation environment, the physical environment) in which people live must be added into already complex models for testing hypotheses. Even in the United States where we have federal commitments [1] and significant investments in biomedical research [4], it is easy to understand why the ideas of new nutrition and food environment–related policies often end up in the discussion sections of scientific manuscripts as a pipe dream but rarely manifest to the point of researchers being able to evaluate an actual policy change.

Blanchard et al. [ ] describe the research currently available at the furthest end of the translational research spectrum; the research that is completed after a policy has been put in place. Specifically, they describe the available scientific literature (i.e., “the best available science and data”) that evaluates “...the effectiveness, cost-effectiveness, development, and implementation, of both regulatory and voluntary policies promoting healthy food environments.” Their analysis reveals that a majority of the research available was investigated only a few specific policy interventions and that the capacity of scientists to do such research only “lies in the hands of a few expert teams…” across the world. This suggests a need for more diversity in *1)* who conducts research on the effectiveness of the policy interventions the entire biomedical research workforce has worked so hard to contribute evidence toward and *2)* the types of policies that are studied. Blanchard et al. also found that even the research that was available, often failed to adequately measure unintended consequences of policy changes. Specifically they found that 50% of studies identified did not look at any changes in health equity, and changes in health equity are common untended consequence of large scale policy changes [5].

Building enough of a reservoir of nutrition and food science research and data on which to move forward even one policy intervention regularly takes years and millions of dollars. Enacting a policy is normally outside of a scientist’s job description and requires a configuration of actions outside of the realm of science and research entirely. However, when a policy is changed it can affect hundreds, thousands, or millions of people and can therefore have major impacts on public health. As a research workforce we should capitalize on these relatively rare and golden opportunities to fully investigate the effects of policy through diversity, as suggested by Blanchard et al. ([ ]. We are charged with generating “the best available science and data” and diversity in thought, in approach, in people, in data, and in geography generates the best nutrition and food-related science and data [[6], [7], [8], [9]].

## Author contributions

The sole author was responsible for all aspects of this manuscript.

## Funding

The authors reported no funding received for this study.

## Conflict of interest

AJV reports a relationship with Academy of Nutrition and Dietetics that includes: travel reimbursement. AJV serves on the editorial board of Advances in Nutrition and the Journal of the Academy of Nutrition and Dietetics. AJV is a federal employee and this manuscript does not reflect the opinion of the federal government.
